# Development of the zebrafish foveal analogue: a quantitative atlas of high-acuity zone growth and retinal regionalisation

**DOI:** 10.1242/bio.062760

**Published:** 2026-09-01

**Authors:** Hammad Syed, Manjiri Patil, William Jakins, Ha-Jun Yoon, Natalie Allcock, Gareth Douglas, William H. J. Norton, Mervyn G. Thomas

**Affiliations:** ^1^ The University of Leicester Ulverscroft Eye Unit, School of Psychology and Vision Sciences, University of Leicester, RKCSB, PO Box 65, Leicester LE2 7LX, UK; ^2^ Department of Neurology, The Johns Hopkins University School of Medicine, Baltimore, MD 21287, USA; ^3^ Electron Microscopy Facility, College of Life Sciences, University of Leicester, Leicester LE1 7HG, UK; ^4^ Hercules Facility, College of Science and Engineering, University of Leicester, Leicester LE1 7RH, UK; ^5^ Division of Biosciences Education, University of Leicester, University Rd, Leicester LE1 7RH, UK; ^6^ Division of Genetics and Genome Biology, University of Leicester, Leicester LE1 7RH, UK; ^7^ Department of Ophthalmology, University Hospitals of Leicester NHS Trust, Leicester LE1 5WW, UK

**Keywords:** High-acuity zone, Fovea, Zebrafish, Retina

## Abstract

The vertebrate retina contains specialised regions for high-acuity vision, exemplified by the human fovea and its zebrafish analogue, the high-acuity zone (HAZ). Despite the widespread use of zebrafish to model retinal disease, a stage-resolved quantitative reference describing normal eye, photoreceptor layer (PRL) and lens growth has been lacking. Here, we apply contrast-enhanced micro-computed tomography (micro-CT) to construct the first three-dimensional micro-CT normative atlas of wild-type zebrafish eye development across five larval stages [3, 5, 7, 10 and 18 days post-fertilisation (dpf)], mapping circumferential PRL thickness, eye and lens morphology, and compartment growth rates. Regional PRL thickening within the temporo-ventral region of the expected HAZ emerged by 5 dpf and was sustained by a localised redistribution of growth, persisting and extending towards the optic nerve through 18 dpf. The PRL, lens and eye grew through four phases, alternating between disproportionate PRL expansion and coordinated growth, while the eye remodelled from a nasal-dominant to a temporo-ventral-dominant form. This regional specialisation was protracted relative to gross ocular growth and could proceed independently of it, paralleling the extended postnatal maturation of the human fovea. This atlas provides a quantitative baseline for distinguishing disease-induced changes from normal variation, supporting zebrafish models of foveal hypoplasia and related disorders.

## Teaser

**Summary:** A three-dimensional atlas of zebrafish eye development, revealing the developmental trajectory of foveal analogue, known as the high-acuity zone, from 3 to 18 days post-fertilisation.

## INTRODUCTION

Retinal specialisation is a conserved feature of the vertebrate visual system (Bringmann et al., 2018; Provis et al., 1998; Rasys et al., 2021; Heukamp et al., 2020; Schmitt and Dowling, 1999). It is characterised by a differential retinal thickness or increase in photoreceptor and/or retinal ganglion cell density in the region (Hughes, 1977). In humans, the macula in the central retina contains the fovea, a specialised region responsible for high-acuity vision (Provis et al., 2013; Thomas et al., 2022). The fovea contains a high density of cone photoreceptor cells and a thick outer nuclear layer (ONL) and is devoid of inner retinal layers (Yamada, 1969). Diseases affecting this region like age-related macular degeneration (AMD), macular dystrophies, and foveal hypoplasia are leading causes of blindness or impaired vision (Gregory-Evans and Gregory-Evans, 2011; Kuht et al., 2022; Fine et al., 2000; Michaelides et al., 2003; Flaxman et al., 2017; Budoff and Poleg-Polsky, 2025; Thomas et al., 2011, 2022). Understanding how specialised retinal regions develop is therefore essential for uncovering the mechanisms underlying these pathologies. Several animal models including mouse, chick, anoles (lizard), birds and non-human primate models have been used for this purpose (da Silva and Cepko, 2017; Peng et al., 2019; Voigt et al., 2019; Sugiyama et al., 2020; Wahle et al., 2023; Rasys et al., 2024). Each has limitations in optical accessibility, throughput, or cost that zebrafish larvae avoid (Zeiss, 2013).

The zebrafish (*Danio rerio*) also offers several advantages over other animal models such as rapid external development, genetic tractability and ease of drug administration. Additionally, the fast functional maturation of the zebrafish visual system (Lieschke and Currie, 2007; Link and Collery, 2015; Schmitt and Dowling, 1999; Bibliowicz et al., 2011), together with a high degree of anatomical and functional similarity to the human retina (Richardson et al., 2017), makes zebrafish a widely used model for modelling retinal diseases (Chhetri et al., 2014).

In most studies involving the use of zebrafish for studying retinal disease, structural phenotypes following gene mutation or drug treatment are generally assessed by quantifying the changes in eye shape and size, lens diameter, retinal layer thickness or cell counts (Caioni et al., 2023; Aguirre-Rebolledo et al., 2025; Avanesov and Malicki, 2010; Mohideen et al., 2003; Quint et al., 2022; Collery et al., 2014; Monfries et al., 2025; Catalano et al., 2007; Li et al., 2012). The interpretability of such mutant and pharmacological studies is limited by the absence of a stage-resolved, quantitative reference atlas describing the normal growth of zebrafish eye, lens and retinal layers across larval stages. Such a reference dataset would also enable direct morphometric comparison of zebrafish retinal development across vertebrate species, providing a strong translational impact.

Of particular relevance to human macular development, the zebrafish retina contains a specialised region, known as the strike zone or the high-acuity zone (HAZ or AZ) (Schmitt and Dowling, 1999; Yoshimatsu et al., 2020). Located in the temporo-ventral region, the HAZ has the highest density of ultraviolet (UV) cones and ganglion cells (Zimmermann et al., 2018; Zhou et al., 2020). HAZ cone cells are long and slender with elongated outer segments (Schmitt and Dowling, 1999), a morphological organisation that bears close functional similarity to the human fovea. This positions the HAZ as an important foveal analogue. Despite its importance, a quantitative developmental trajectory of the HAZ across larval stages remains lacking, limiting its use as a comparative model of human macular disease.

To address this gap, we selected key developmental stages based on the established temporal sequence of zebrafish retinal maturation. The zebrafish retina develops by ∼3 days post-fertilisation (dpf) including the establishment of basic synaptic connections, extraocular muscles and laminated architecture (Schmitt and Dowling, 1999; Easter and Nicola, 1996). By 3 dpf, the shape of the lens becomes spherical in all dimensions, suggesting a functional optical element (Wang et al., 2020; Greiling and Clark, 2009). Functionally, the first visual-evoked responses are seen by 3 dpf (Easter and Nicola, 1996), with a robust optokinetic response observed as early as 5 dpf (Neuhauss, 2003). At early stages, retinal differentiation occurs as a spatial patterning wave starting from the ventral patch, extending to the nasal, dorsal, and finally temporal retina (Raymond et al., 1995; Schmitt and Dowling, 1996; Neumann, 2001; Easter and Nicola, 1996). By 5 dpf, all photoreceptor subtypes in the zebrafish retina – red, green, blue and UV – are morphologically discernible (Branchek and Bremiller, 1984; Crespo and Knust, 2018). Maturation of the cone subtypes occurs in a staggered fashion, with the long single blue cones present at 7-8 dpf and the paired red and green cones becoming distinct by 10-12 dpf (Branchek and Bremiller, 1984; Raymond et al., 1995). Rod photoreceptor cells, although detected early, become mature between 15 and 21 dpf, with the zebrafish exhibiting adult-like electroretinogram by 21-24 dpf (Saszik et al., 1999). Together, these developmental milestones informed the selection of five stages (3, 5, 7, 10, and 18 dpf) spanning the onset of visual function to near-mature retinal physiology.

A major obstacle to studying retinal growth and HAZ development is the lack of imaging modalities capable of providing a comprehensive, quantitative measurement at the level of the whole eye. While a few impressive confocal-based studies have illustrated the early development of the HAZ in the context of cone and ganglion cell density (Yoshimatsu et al., 2020; Hernández-Bejarano et al., 2022; Tarboush et al., 2012), a complete trajectory of its formation and growth still needs to be studied. Conventional histological sectioning provides an accurate representation of the retinal lamination and thickness; however, it is low throughput and limited to a few sections per eye. This is particularly challenging for the HAZ, which is captured only in a few sections and requires correct orientation across samples for unbiased comparison (Fig. S1A,B). Confocal microscopy, while powerful for cellular resolution imaging, is limited by pigmentation in the retinal pigment epithelium (RPE) and has mostly been applied to young larvae. High-resolution techniques like electron microscopy are best suited for looking at finer structure and are not suited for whole-eye reconstruction.

Micro-computed tomography (micro-CT) offers a complementary approach that addresses these limitations directly. Micro-CT is unaffected by RPE pigmentation and enables non-destructive, isotropic three-dimensional reconstruction of the whole eye, rather than measurement from a few reference sections. Although traditionally applied to mineralised tissue (Liao et al., 2023; Hayes et al., 2014; Seo et al., 2015), contrast agents such as iodine and phosphotungstic acid staining have extended its use to soft tissues (Babaei et al., 2016; Weinhardt et al., 2018), including whole-body imaging of adult zebrafish and medaka (Katz et al., 2021).

In this study, we apply contrast-enhanced micro-CT to construct the first three-dimensional normative atlas of wild-type (WT) zebrafish eye development across five key larval stages (3, 5, 7, 10 and 18 dpf), quantifying eye morphology, lens dimensions, and compartment growth rates. Throughout the study, photoreceptor layer (PRL) thickness serves as a structural proxy for HAZ specialisation. By providing both a stage-resolved growth reference and a quantitative account of how the HAZ emerges and is maintained, this atlas addresses two outstanding gaps in our knowledge of zebrafish visual-system development: (1) the absence of a normative growth baseline and (2) the limited quantitative understanding of HAZ formation in the context of PRL thickness.

## RESULTS

### Regional specialisation of PRL thickness during larval development

To characterise the emergence of the regional specialisation in the PRL, we mapped PRL thickness across the full retinal circumference at five stages spanning early to late development (3, 5, 7, 10 and 18 dpf). Maximum thickness values were compared across three retinal regions: the temporo-ventral quadrant (corresponding to the HAZ) and the dorsal and nasal retina (see Materials and Methods for details). This comparison was used to determine the spatiotemporal location and extent of regional PRL thickening.

**Fig. 1.**
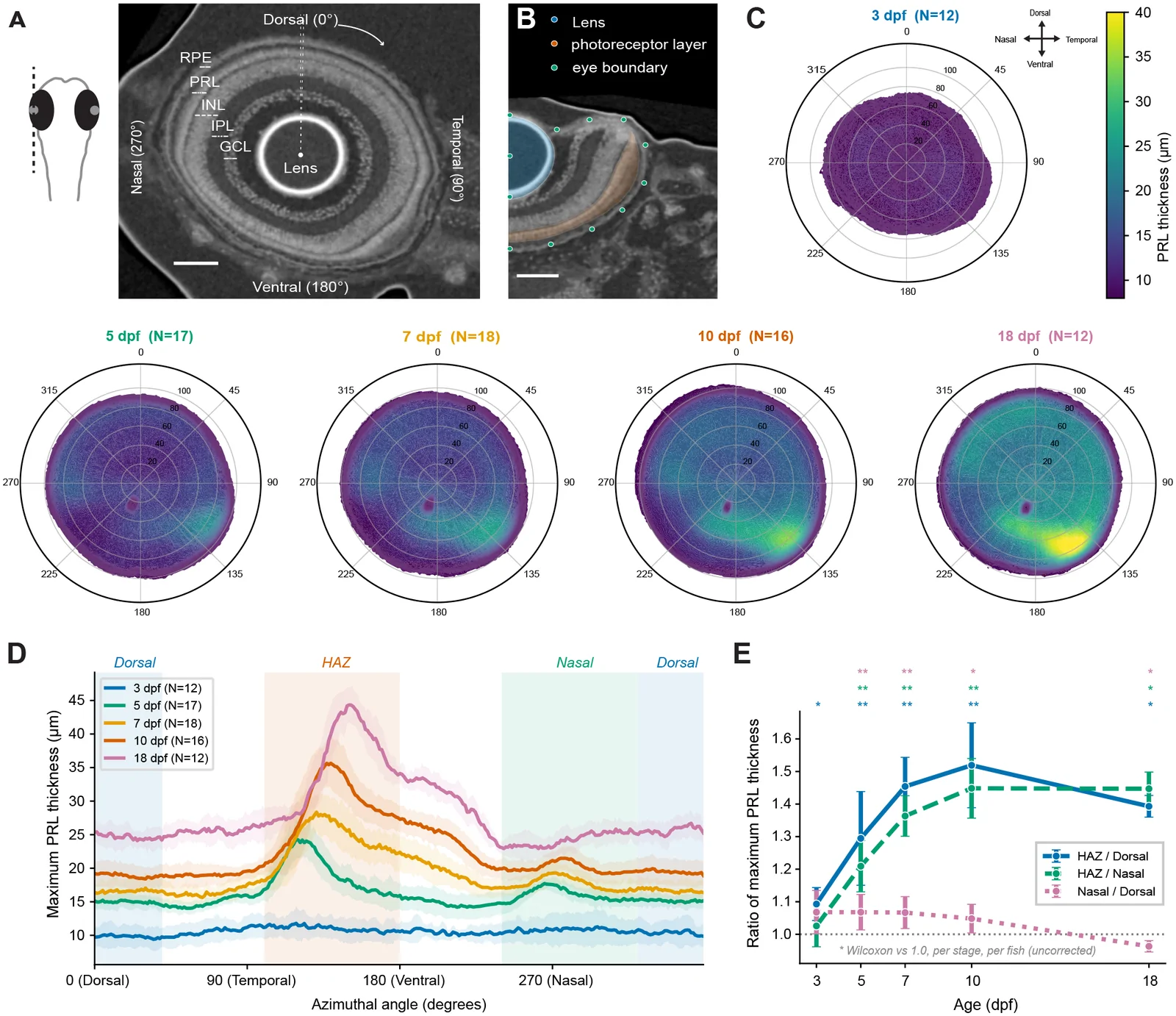
**PRL thickness across developmental stages.** (A) Sagittal micro-CT cross section of a WT zebrafish left eye at 5 dpf. Retinal layers and anatomical orientations are labelled. Representative lines to obtain radially resliced section are shown with white dashed lines. Scale bar: 50 µm. (B) A representative radially resliced section with segmentation masks overlaid: lens (blue), PRL (orange), and eye boundary landmarks (green). Scale bar: 50 µm. (C) Polar flatmaps of PRL thickness for WT at 3, 5, 7, 10 and 18 dpf. Each map represents the distribution of PRL thickness averaged over the number of eyes per stage. Radial coordinate (20, 40, 60, 80, 100) represents the ray index from the lens centre. Together, radial and azimuthal angles (0-360°) show the thickening of the PRL in the temporo-ventral quadrant. Colour encodes local PRL thickness (µm; viridis scale, 8-40 µm shared across all stages). The compass rose indicates anatomical orientation. (D) Azimuthal profile plot of maximum PRL thickness across the eye at each developmental stage. Lines represent stage means; shaded bands indicate ±1 s.d. Vertical shaded regions denote the HAZ (orange; 100-180°), nasal (green; 240-320°), and dorsal (blue; 320-40°) reference zones. *N* indicates the number of eyes per stage. (E) Ratios of maximum PRL thickness between anatomical zones, calculated per fish as the mean of paired left and right eyes and averaged across animals within each stage (*N*=6-9 fish per stage). HAZ/dorsal (blue, solid), HAZ/nasal (green, dashed), and nasal/dorsal (pink, dotted) ratios are shown as mean±s.d. Dashed reference line (black) at 1.0 indicates equal thickness between zones. Asterisks indicate ratios significantly different from 1.0 (Wilcoxon signed-rank test, per stage, uncorrected; *P<0.05, **P<0.01). RPE, retinal pigment epithelium; PRL, photoreceptor layer; INL, inner nuclear layer; IPL, inner plexiform layer; GCL, ganglion cell layer; HAZ, high-acuity zone.

At 3 dpf, the maximum PRL thickness was approximately uniform throughout the retina (Fig. 1C,D). The absolute maximum thickness values were comparable across the three zones (HAZ: 11.03±1.36 µm; dorsal: 10.11±1.30 µm; nasal: 10.77±1.31 µm). By 5 dpf, the maximum PRL thickness in the temporo-ventral retina significantly exceeded both the dorsal and nasal zones (HAZ-to-reference zones ratio>1.0, *P*<0.01). Maximum PRL thickness in the HAZ reached 19.40±1.72 µm, compared with 15.03±0.81 µm in the dorsal zone and 16.03±0.71 µm in the nasal zone. A secondary region of elevated PRL thickness was also apparent in the nasal retina (∼250-300°), though less prominent than the HAZ (Fig. 1C,D,E).

By 7 dpf, PRL thickness in the HAZ (24.14±2.14 µm) was 1.45-fold greater than that in the dorsal retina and 1.36-fold greater than that in the nasal retina. As the eye grew, the HAZ PRL thickness increased progressively throughout all developmental stages [Mann-Whitney *U* test with Benjamini-Hochberg (BH) correction, *P*<0.001 for all consecutive comparisons except at 7-10 dpf, *P<*0.01] and spread towards the optic nerve (∼200°, see Materials and Methods) between 10 and 18 dpf. By 18 dpf, the maximum PRL thickness reached 35.04±0.78 µm. Notably, the elevated PRL thickness observed in the nasal region at 5 dpf remained through 10 dpf (nasal-to-dorsal ratio>1.0, *P*<0.05) but was lost by 18 dpf (Fig. 1E). The dorsal PRL thickness exceeded nasal thickness by this stage (*P<*0.05).

The spatial pattern of regionalisation was consistent when assessed using mean rather than maximum PRL thickness, with the HAZ remaining the thickest region at all stages from 5 dpf onwards (Fig. S1C). Together, these results demonstrated progressive and spatially restricted thickening of the PRL in the HAZ from 5 dpf onwards, establishing it as a prominent zone of retinal specialisation through 18 dpf.

### Differential growth of the PRL and lens during eye development

Having established that the PRL becomes regionally thickened from 5 dpf onwards, we next asked whether this thickening is achieved by a disproportionate growth of the PRL relative to the whole eye or a coordinated growth of eye compartments. To address this, we compared the volumetric growth of the PRL, lens, and eye across developmental stages and calculated specific growth rates (SGRs) between consecutive stages as a descriptive summary of volumetric change. We also evaluated the PRL and eye sector-wise growth rates to determine regionally distinct growth patterns between different stages.

**Fig. 2.**
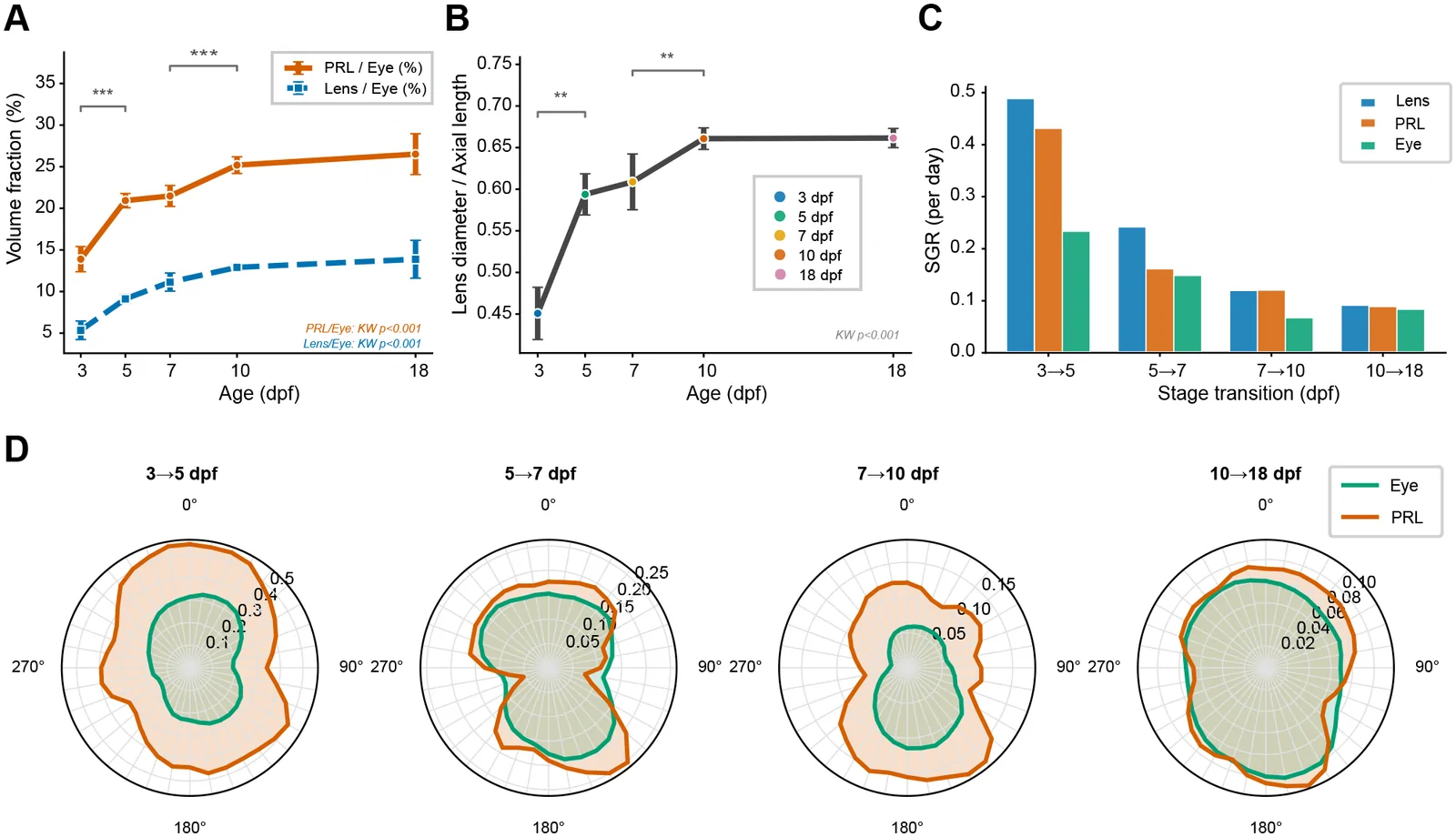
**Growth of the PRL, lens and eye across developmental stages.** (A) Volume fraction of the PRL (orange, solid line) and lens (blue, dashed line) expressed as the percentage of the total eye volume. Data points represent mean±s.d. per fish (*N*=6-9 fish per stage). Significance brackets indicate stages with significant pairwise differences in the PRL/eye fraction (Mann-Whitney *U* test, BH corrected; **P<0.01, ***P<0.001). Significance brackets for the lens/eye fraction are not shown here. (B) The ratio of lens diameter to eye axial length per fish across developmental stages. Data points represent mean±s.d. per stage (*N*=6-9 fish per stage). (C) SGR (day^−1^) of the lens (blue), PRL (orange), and whole eye (green) across four consecutive stage transitions. The SGR was calculated as (lnV_2_−lnV_1_)/Δt from stage mean volumes and is shown for descriptive purposes. (D) Sector-level SGR of the PRL (orange) and eye (green) visualised as overlaid radar charts across four stage transitions (3→5, 5→7, 7→10, 10→18 dpf). Each of the 36 spokes represents a 10° azimuthal sector. Values plotted are absolute SGR (day^−1^). The radial axis maximum is scaled independently per panel. Orientation follows the left-eye convention: dorsal=0°, temporal=90°, ventral=180°, nasal=270°. PRL, photoreceptor layer.

We found that the progressive thickening of the PRL in the HAZ is accompanied by a disproportionate increase in PRL volume relative to the whole eye. At 3 dpf, PRL occupied only 13.89±1.51% of the total eye volume, while the lens took up 5.34±1.11%. Between 3 and 5 dpf, the PRL volume grew significantly relative to the whole eye, reaching 20.93±0.86% (*P*<0.001). The SGR was nearly twice as high for the PRL (0.430 day^−1^) as for the whole eye (0.232 day^−1^) during this period (Fig. 2C).

During the 5-7 dpf time period, the PRL and whole eye grew roughly at the same rate (Fig. 2A,C), with no significant change in the PRL-to-eye ratio (20.93±0.86% versus 21.48±1.26%, respectively, *P=*0.25). A second phase of disproportionate PRL expansion was observed between 7 and 10 dpf, with the PRL-to-eye ratio increasing significantly from 21.48±1.26% at 7 dpf to 25.19±1.00% at 10 dpf (*P*<0.001), consistent with a higher PRL SGR (0.119 day^−1^) relative to the eye (0.066 day^−1^).

Additionally, the lens-to-eye volume ratio showed a significant increase at every transition from 3 to 10 dpf, with no plateau (all *P*≤0.01, BH corrected), contrasting with the PRL-to-eye ratio. Interestingly, the ratio of lens diameter to axial length showed a two-phase pattern similar to PRL growth. The ratio of lens diameter to the axial length of the eye increased significantly at 3-5 and 7-10 dpf (both *P*<0.01, BH corrected), while it remained unchanged at 5-7 and 10-18 dpf (Fig. 2B). This two-phase pattern in the ratio of lens diameter to axial length contrasts with the continuous increase in lens volume fraction across the same period, suggesting that lens volumetric growth and lens diameter growth relative to axial length are not tightly coupled during early larval development.

During the last developmental transition period, all the compartments achieved a coordinated growth rate with no significant increase in the PRL-to-eye or lens-to-eye ratio from 10 to 18 dpf (Fig. 2A). This convergence corresponds to the period when the HAZ-to-nasal and HAZ-to-dorsal ratios reach a plateau (Fig. 1E), highlighting the transition from a period of disproportionate growth at early stages to a more coordinated growth at later stages.

In addition to the disproportionate growth during early stages of development, the PRL itself undergoes differential growth in different sectors of the eye at each developmental transition, as shown in the sector SGR heatmap (Fig. 2D). During 5-7 dpf, when the PRL and eye grew proportionately, the SGR was highest in the PRL region, overlapping with the HAZ (100-180°) compared with other regions. This pattern of faster temporo-ventral PRL growth was apparent through 7-10 and 10-18 dpf (Fig. 2D).

Together, these results reveal alternating phases of disproportionate and coordinated growth in both the PRL volume relative to the whole eye and lens diameter relative to the axial length, with a sustained temporo-ventral dominance at the sector level from 5 dpf onwards. These differential growth dynamics underlie the emergence and maintenance of HAZ regionalisation in the PRL.

### Remodelling of eye shape accompanies retinal regionalisation

We next asked whether the eye itself undergoes asymmetric remodelling that accompanies or allows retinal specialisation. For this, we assessed eye shape using three ratios, dorsal/ventral (D/V), temporal/nasal (T/N), and dorso-ventral/naso-temporal (DV/NT), to identify systematic shifts in eye geometry across development.

We found that the eye grew substantially across larval development. The maximum diameter increased from 270.95±13.85 µm at 3 dpf to 428.28±14.07 µm at 18 dpf, and its shape underwent progressive remodelling across this period. All three ratios changed significantly across the five developmental stages [Kruskal-Wallis (KW), *P*<0.001 for all].

**Fig. 3.**
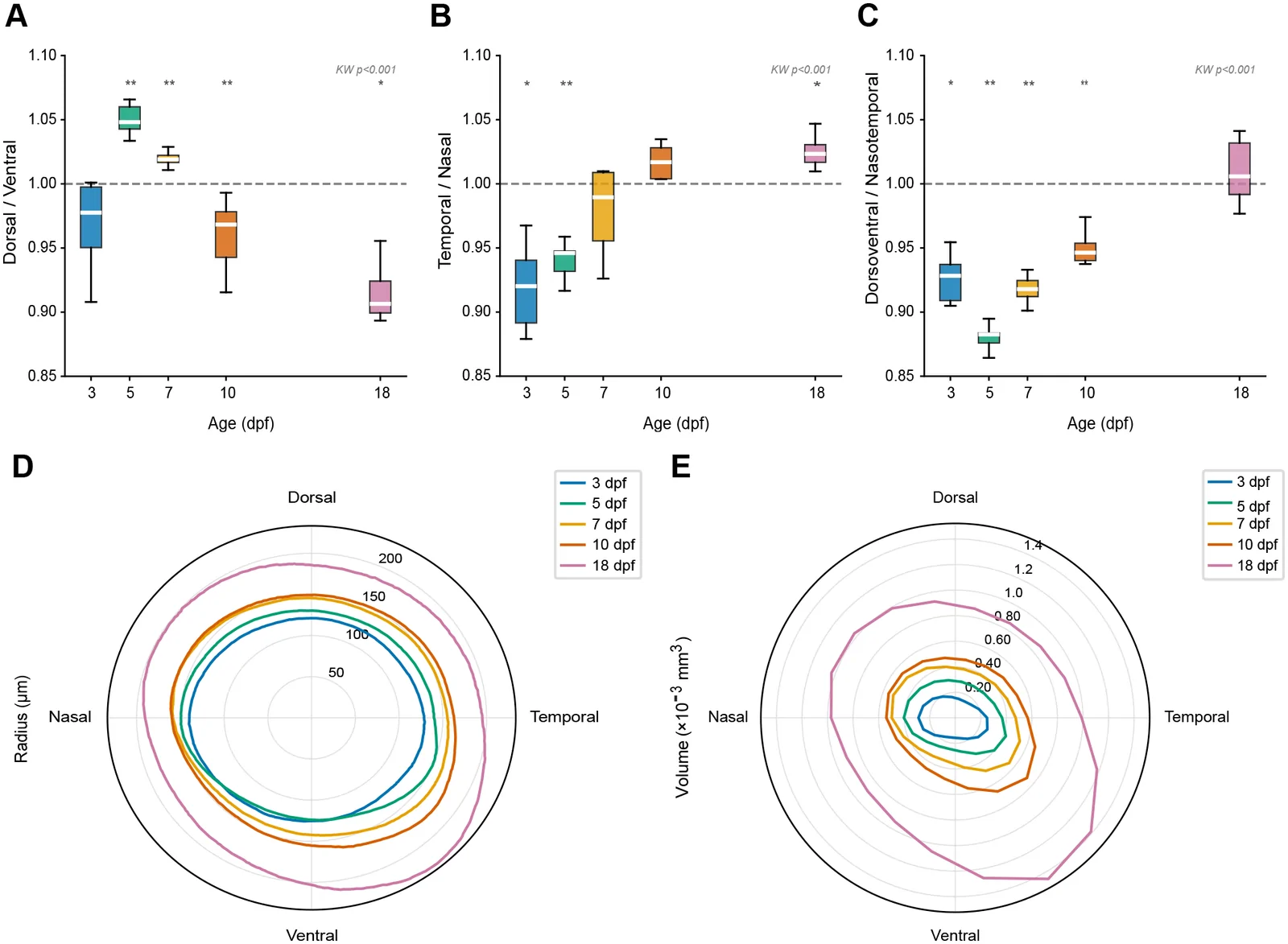
**Eye morphology across developmental stages.** (A) D/V ratio of the eye radius across developmental stages. (B) T/N ratio of the eye radius across developmental stages. (C) DV/NT diameter ratio across developmental stages. For panels A-C, boxplots show per-fish distribution (*N*=6-9 fish per stage). The white line indicates the median. Asterisks indicate ratios significantly different from 1.0 (Wilcoxon signed-rank test, per stage, uncorrected; *P<0.05, **P<0.01). (D) Circumferential radius profiles of the eye at each developmental stage, displayed as stage-mean polar plots. Each line represents the mean radial distance from the lens centre to the eye boundary across all 360° azimuthal positions. Orientation follows the left-eye convention: dorsal=0°, temporal=90°, ventral=180°, nasal=270°. *N* indicates the number of fish per stage. Radial axis in µm. (E) Regional distribution of eye volume across azimuthal sectors at each developmental stage. Each polygon represents the stage-mean eye volume (×10^−3^ mm^3^) per 20° sector across 18 sectors spanning the full 360° of the eyes.

At 3 dpf, the eye was roughly symmetric in the DV axis (D/V mean 0.968, *P=*0.09). At 5 dpf, the dorsal radius became significantly greater than the ventral radius (mean 1.056, *P<*0.01) and remained so through 7 dpf (1.021, *P<*0.01). However, by 10 dpf, the ventral radius exceeded the dorsal radius (mean 0.961, *P*<0.01), and this ventral dominance persisted through 18 dpf (mean 0.915, *P<*0.05) (Fig. 3A).

The T/N ratio showed a gradual shift across larval development. The nasal radius remained dominant over the temporal radius through 3 dpf (mean 0.919, *P*<0.05) and 5 dpf (mean 0.937, *P*<0.01). At 7 dpf, the temporal and nasal radii became similar (mean 0.980, *P*=0.16) and remained so at 10 dpf (mean 1.020, *P*=0.15). By 18 dpf, the temporal radius became significantly greater than the nasal radius (mean 1.025, *P<*0.05) (Fig. 3B).

The DV diameter remained smaller than the NT diameter throughout 3-10 dpf, with the DV/NT ratio remaining significantly below 1.0 (3 dpf: mean 0.917, *P<*0.05; 5-10 dpf: range 0.881-0.945, all *P<*0.01). By 18 dpf, the two diameters converged with no significant difference between them (mean 1.010, *P=*0.56) (Fig. 3C).

The circumferential radius profile (Fig. 3D) shows the full eye shape at each developmental stage. The regional distribution of eye volume across azimuthal sectors is shown in Fig. 3E, illustrating progressive expansion in the ventral and temporal regions. Across the developmental period examined, eye shape transitioned from a nasal-dominant, dorso-ventrally symmetric form at 3 dpf to a temporal- and ventral-dominant form by 18 dpf, consistent with the progressive expansion of the temporo-ventral retina observed alongside HAZ development.

## DISCUSSION

In this study, we used micro-CT imaging to generate a quantitative micro-CT normative atlas of zebrafish eye, PRL, and lens growth across five larval stages spanning early to late development. We identified three principal features of the developing larval visual system: (1) regional thickening of the PRL used as a structural proxy for HAZ specialisation within the temporo-ventral retina by 5 dpf, sustained by a redistribution of growth; (2) four distinct phases of PRL compartment growth; and (3) a progressive asymmetric remodelling of the eye that accompanies HAZ expansion. Below, we discuss the developmental and functional implications of these findings.

PRL thickening in the HAZ emerges by 5 dpf and continues to develop through the latest stage examined (18 dpf). Consistent with early electron microscopy and immunostaining observations (Schmitt and Dowling, 1999; Yoshimatsu et al., 2020; Hernández-Bejarano et al., 2022), we detect the emergence of HAZ specialisation by 5 dpf, the stage at which larvae initiate foraging activities and active prey capture (Yoshimatsu et al., 2020). The elevated PRL thickness in this region is consistent with the increased density of UV cone photoreceptors and longer outer-segment length in the HAZ observed by 5 dpf, which are needed for detecting paramecia on the water surface (Lahne et al., 2026 preprint; Westö and Ala-Laurila, 2020; Novales Flamarique, 2016, 2013; Zimmermann et al., 2018). Our circumferential mapping shows that this regionalisation is spatially specific, achieved by a relatively high growth in the temporo-ventral quadrant. Specifically, HAZ-to-dorsal and HAZ-to-nasal ratios remained significantly greater than 1.0 at all stages from 5 dpf, while the nasal-to-dorsal ratio remained close to 1.0 throughout, indicating that the two reference zones grow in parallel. Between 10 and 18 dpf, the region of elevated PRL thickness extended ventrally towards the optic nerve. The cellular basis of this later expansion remains to be determined, as outer-segment growth and increased packing efficiency in the HAZ have so far been reported only up to 5 dpf (Lahne et al., 2026 preprint; Hunt et al., 2026 preprint).

The PRL, lens and eye do not grow at uniform rates but pass through four phases of relative expansion. The phase boundaries are grounded in the per-fish ratios of PRL to eye and lens diameter to axial length, while the SGRs provide a complementary illustration of the underlying volumetric changes descriptively. In Phase I (3-5 dpf), the PRL grows disproportionately faster relative to the eye. This coincides with the reported period of major morphological maturation of the photoreceptor compartments (inner segment, outer segment, cell body) that gives rise to the first robust visual response by 5 dpf (Crespo and Knust, 2018; Neuhauss, 2003).

In Phase II (5-7 dpf), the PRL and eye grew at a similar overall rate, with no change in the PRL-to-eye ratio. Notably, the ratio of lens diameter to axial length also remains stable across this window, indicating that the overall optical geometry is held approximately constant during this phase. However, HAZ-to-nasal and HAZ-to-dorsal ratios continue to increase, with growth concentrated in the temporo-ventral area as shown descriptively by the sector-level SGR (Fig. 2D). This indicates that regional PRL thickening in the HAZ can proceed without net expansion of the PRL relative to the eye. This dissociation between global and regional PRL growth may have practical implications for zebrafish models of foveal hypoplasia: a normal overall PRL size does not exclude a regional HAZ patterning defect, and conversely, a reduction in total PRL volume does not necessarily imply loss of HAZ specialisation. Resolving these distinctions may be aided by a circumferential mapping achievable by micro-CT 3D imaging, in conjunction with histological or confocal measurements.

Phase III (7-10 dpf) marks a second phase of disproportionate PRL growth, with a significant increase in the PRL-to-eye ratio. This temporal window coincides with the reported cone outer-segment elongation, which continues until approximately 15 dpf (Branchek and Bremiller, 1984), and with the transition from a single layer to a stratified photoreceptor arrangement from around 8 dpf (Hitchcock and Raymond, 2004). Both processes would be expected to increase PRL thickness, although direct measurement of outer-segment length and photoreceptor layering would be required to confirm their contribution.

Phase IV (10-18 dpf) is a period of coordinated growth: PRL, lens, and eye growth rates converged to equivalent values; the ratio of lens diameter to axial length stabilised, and the HAZ-to-dorsal and HAZ-to-nasal ratios plateaued. This coincides with the completion of cone outer-segment maturation and the approach of anatomical rod maturity (Branchek and Bremiller, 1984), suggesting that Phase IV likely represents a transition from active photoreceptor morphogenesis to maintenance of the established retinal architecture.

Lens growth presents two distinct trajectories depending on the readout used. By volume, the lens-to-eye ratio increases continuously across every transition from 3 to 10 dpf. This continued increase in lens volume fraction is consistent with the lens's role as a reference structure for body and eye size, with the need for ongoing accumulation of lens fibre material as the eye expands (Greiling and Clark, 2009). By optical geometry, however, the ratio of lens diameter to axial length follows a phased pattern similar to PRL. It increases significantly during 3-5 and 7-10 dpf (Phases I and III) and remains unchanged during 5-7 and 10-18 dpf (Phases II and IV). This phased trajectory of lens diameter to axial length determines where the image plane sits relative to the PRL (Collery et al., 2014). The convergence of this trajectory is consistent with the trend observed for the HAZ-to-reference zone ratio and PRL-to-eye ratio and likely represents the stabilisation of the visual system.

This is accompanied by remodelling of the overall eye shape. The eye transitioned progressively from nasal dominant at 3 dpf to temporal dominant by 18 dpf, with the ventral radius becoming progressively larger from 10 dpf onwards. The NT and DV asymmetry of the eye shape is consistent with the structural expansion of the temporo-ventral retina, accommodating the growing HAZ. These asymmetric changes in ocular globe size during eye development are a common feature observed in a number of foveated species (Rasys et al., 2024). The structural expansion of the temporo-ventral retina is also ecologically important. As the fish grows, foraging depth and prey size diversify (Sabatés and Saiz, 2000; Pepin, 2024), and continued spatial expansion of the eye alongside the HAZ accommodates the increasing demands placed on the high-acuity visual system.

**Fig. 4.**
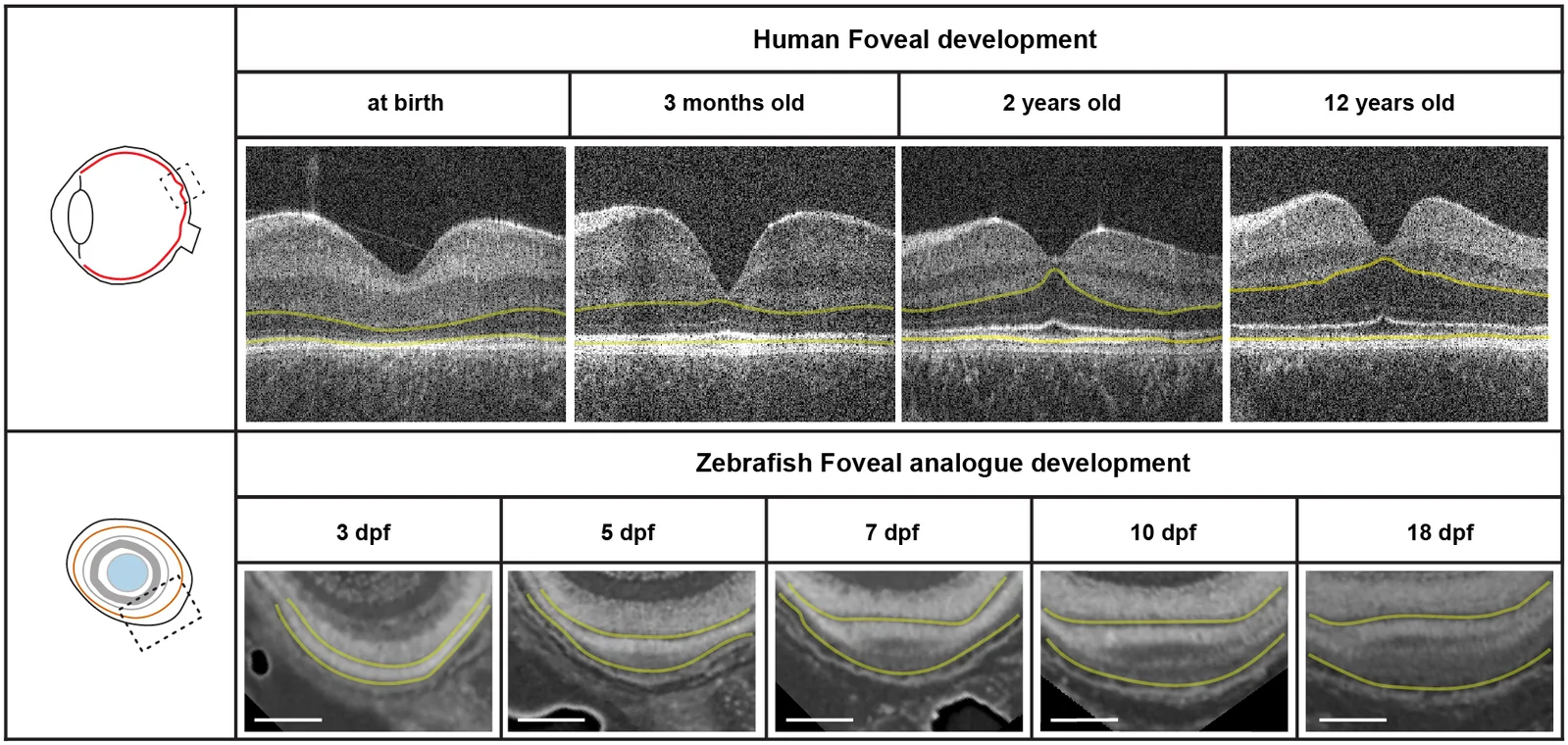
**Thickening of the PRL accompanies foveal and HAZ development in human and zebrafish retina.** (Top) OCT B-scans of the human fovea at birth, 3 months, 2 years, and 12 years of age, illustrating the progressive postnatal maturation of the central retina. (Bottom) Micro-CT sagittal sections of zebrafish foveal analogue at 3, 5, 7, 10, and 18 dpf. Scale bars: 50 µm. The PRL boundary is highlighted in yellow in both. The two rows indicate a shared developmental principle and are not intended to imply temporal equivalence between given human and zebrafish developmental stages. Schematics (left) show the approximate plane of the section through the human retina (top) and zebrafish left eye (bottom).

Although the zebrafish lacks a true foveal pit and a foveal avascular zone, the structural logic by which the HAZ is constructed parallels that of the human fovea. In humans, the central retina is specified early as a cone-only, ganglion-cell-rich patch, but the hallmarks of high-acuity specialisation, namely, cone outer-segment elongation, narrowing of the cone diameter and increased cone packing density with consequent thickening of the outer nuclear and Henle fibre layers, are acquired largely postnatally and continue for years after the onset of vision (Hendrickson and Yuodelis, 1984; Hendrickson et al., 2012; Provis et al., 2013; Thomas et al., 2022). Foveal outer-segment length and cone packing density remain only about half their adult values at 45 months, well after the eye has become functional (Yuodelis and Hendrickson, 1986). In the zebrafish HAZ, increased cone outer-segment length and cone packing density have been directly demonstrated up to 5 dpf (Lahne et al., 2026 preprint; Hunt et al., 2026 preprint), and our data show that regional PRL thickening continues well beyond this point, extending ventrally through 18 dpf (Fig. 4). Whether the same cellular processes drive this later thickening remains to be determined, but the continuity of the thickening trajectory is consistent with an extension of these mechanisms. In both systems, regional specialisation is protracted relative to gross ocular growth and can proceed even when the PRL is not expanding relative to the whole eye (Phase II), highlighting that high-acuity specialisation is a locally regulated process rather than a by-product of global eye enlargement. These parallels carry translational value, since ONL widening and outer-segment lengthening, the structural correlates used to grade foveal development in foveal hypoplasia (Thomas et al., 2011), are the same features that distinguish the specialised zebrafish HAZ.

The normative atlas presented here provides a quantitative reference value for PRL thickness, eye morphology, lens dimensions, and compartment volumes at five developmental stages (Tables S4 and S5). This dataset enables a direct comparison with future studies using mutant lines, pharmacological treatments, or disease models affecting retinal or eye development. The combination of overall and sector-resolved 3D metrics allows the atlas to discriminate between mechanistically distinct phenotypes at a level that is not possible using conventional techniques. In conditions such as retinitis pigmentosa (Santhanam et al., 2020; Zhang et al., 2021), AMD (Noel et al., 2020), and foveal hypoplasia, where the direct effect on the PRL is expected, the normal thickness distribution across the retina at key developmental stages will be very useful. The micro-CT pipeline is also extensible to other small vertebrate eye models and to additional zebrafish genetic backgrounds and developmental windows.

Several limitations should be noted. The atlas was generated using only the AB strain of zebrafish; however, modest strain-related differences in absolute measurements cannot be excluded. Tissue processing for micro-CT, including fixation, dehydration and resin embedding, may produce some shrinkage of absolute dimensions. We were not able to assess per-layer shrinkage or determine whether any such change is isotropic since micro-CT and comparative techniques like histological sectioning are mutually exclusive terminal procedures and cannot be applied to the same fish. Published work using a broadly similar contrast-enhanced micro-CT preparation on zebrafish has, however, reported cellular dimensions that match independent published values (Ding et al., 2019), suggesting that our sample preparation would preserve cellular-scale morphology well. Absolute measures reported here should nonetheless be interpreted as specific to this preparation, and cross-modality comparison of absolute dimensions should be done carefully. However, as all specimens underwent identical processing, the ratio- and trajectory-based conclusions presented here are robust to uniform scaling. Eye shape asymmetry was quantified using four cardinal coordinates; the full circumferential radius profile, provided in Fig. 3D, offers a more complete description of shape and should be used for fine-scale analyses.

We anticipate that this atlas will serve as a reference standard for studies of retinal degeneration, cataract, myopia, and foveal specialisation in zebrafish, enabling researchers to distinguish disease-induced structural changes from normal developmental variation and accelerating the use of zebrafish as a quantitative platform for ocular disease research.

## MATERIALS AND METHODS

### Animals

WT AB strains were maintained at 28.4°C under a light/dark cycle of 14 h/10 h. Adults were set up as group crosses (two males and two females per tank) overnight with a mesh divider and spawning tray. Breeding was initiated by removing the divider during the light cycle, and the fertilisation time was noted. The fertilised eggs (30-40) were collected in 100 mm Petri plates containing fish water and grown in an incubator set at 28.5°C, 14-h light:10-h dark cycle until 5 dpf. After 5 dpf, they were transferred to an aquarium rack system and fed thrice daily with Zebrafeed (by Sparos) until 18 dpf.

Healthy fish were euthanised with tricaine methanesulfonate MS-222 (328 mg/l) prior to fixation at each time point. Fish at each developmental stage were sampled from two to four clutches, with no single clutch contributing more than half of the individuals at any stage. A total of 38 fish were used across five developmental stages: 3, 5, 7, 10, and 18 dpf. Both left and right eyes were used for analysis, yielding a final dataset of 75 eyes (3 dpf: *N*=12; 5 dpf: *N*=17; 7 dpf: *N*=18; 10 dpf: *N*=16; 18 dpf: *N*=12).

### Ethics

All procedures were performed in the Preclinical Research Facility at the University of Leicester and conducted under Project Licence PP1567795 in accordance with the Animals (Scientific Procedures) Act 1986, with individual researchers holding appropriate personal licences.

### Tissue preparation and micro-CT imaging

Fish samples were fixed in 10% formalin for 24 h at room temperature. They were washed with 1× PBS and placed in 70% ethanol for 1 h. After this step, the samples were stained with a contrast reagent, 0.3% w/v phosphomolybdic acid (Sigma-Aldrich) in 70% ethanol overnight. They were dehydrated through a series of ethanol washes: 90% ethanol for 20 min and 100% ethanol for 30 min twice. They were then washed twice in propylene oxide (PO, Sigma-Aldrich) for 5 min each to remove any residual ethanol. The samples were then infiltrated through a graded (3:1, 1:1, 1:3; v/v) PO:Spurr's modified resin washes for 90 min each before leaving them in fresh resin overnight. All the steps were performed at room temperature on a rotor. The samples were clamped on reverse forceps for polymerisation at 60°C for 16 h in an oven. Finally, six to nine samples were mounted on a carbon rod for micro-CT imaging.

Micro-CT imaging was performed on a ZEISS Xradia CrystalCT with the following settings: 40 kV voltage, 74 µA current, 4501 projections/360°, five-frame averaging with 1-s exposure per frame, using a flat panel complementary metal-oxide-semiconductor detector. The mounted samples were imaged with a 1.0 µm isotropic voxel size in stitch mode to expand the axial field of view, enabling imaging in a single acquisition. A standard Feldkamp, Davis, and Kress algorithm was used to reconstruct the radiographic images to produce a 3D volume file using the manufacturer's software (Scout-and-Scan Control System, ZEISS).

Raw TIFF stacks were imported into Fiji (ImageJ, NIH) for manual orientation alignment (Schindelin et al., 2012). Each fish was rotated and repositioned to provide an initial standardisation of the DV and NT axes across all samples, using the optic nerve as a consistent anatomical landmark to orient the image stack. Aligned sagittal stacks were radially resliced from a reference line drawn from the lens centre to the dorsal retina (0°), proceeding clockwise through 360° in 1° increments to generate 360 cross-sectional slices per eye. The right eye was resliced in the mirror orientation relative to the left eye, such that dorsal corresponds to 0°, temporal to 90°, ventral to 180°, and nasal to 270°, consistent with the left-eye convention when viewed from the anterior aspect. The position of the optic nerve head was assessed manually using the raw images and the obtained segmented masks (see ‘Image segmentation’ section), and all eyes and their corresponding masks were circularly shifted by a common offset to bring the optic nerve head at ∼200°, correcting for residual angular offsets from the orientation step and standardising its position across samples.

### Image segmentation

A custom model was trained on radially resliced images on ZEISS arivis Cloud for semantic segmentation of the lens and the PRL (Carl Zeiss Microscopy GmbH, 2025, https://www.arivis.cloud). The PRL comprised the cell body, inner segment and outer segment of the photoreceptor cells identified by their differential staining. The training network used an EfficientNet-B0 encoder with a U-Net decoder and was applied to 16-bit greyscale image tiles of 192×192 px with a 24 px tile overlap. The dataset consisted of 77 manually annotated images spanning five developmental stages and multiple quadrants of the eye, including 46 images from morphologically distinct mutant larvae to increase training diversity (Table S1). During training, arivis Cloud partitioned these data into internal training and validation subsets, and training/validation loss, pixel accuracy and mean IoU were monitored to convergence (Fig. S2). Segmentation performance for the PRL and the lens was evaluated on a held-out test set of 12 images spanning all five developmental stages (3-18 dpf) (Tables S2 and S3).

DeepLabCut (DLC, 2.3.9) was used to train a custom model for extracting the eye boundary using a ResNet-50 architecture pretrained on ImageNet (Mathis et al., 2018). DeepLabCut was selected for its robust key point localisation and augmentation pipeline, allowing consistent marking of the eye periphery across varying sizes and image quality (Nath et al., 2019). The network was configured to predict 12 key points (E1-E12) along the eye periphery to capture its outline (Fig. 1B). A total of 200 frames were manually labelled spanning the five developmental stages (20 frames from each of the two eyes from different fish) and were used for training, with a 95%/5% train/test split. Training was performed using stochastic gradient descent with DLC's multistep learning-rate schedule (0.005 for 10,000 iterations and then 0.02) and imgaug augmentation (rotation, crop, emboss, scale, contrast adjustment) and was run for 100,000 iterations, with a final loss converging at 0.0033 (Fig. S3). Localisation accuracy, measured as the mean Euclidean distance between predicted and annotated key points, was 1.54 px (train) and 1.61 px (test), at 1 µm/px. The trained network was applied to all 360 azimuthal (1°) reslices of each eye.

Eye masks were generated by connecting DeepLabCut key point coordinates as a closed polygon, while lens and PRL masks were obtained from the arivis Cloud segmentation model. Segmented masks were inspected manually on DragonFly software (v2022.2.0.1409) before analysis.

### PRL thickness measurement

PRL thickness was calculated using a Euclidean distance transform applied to the binarised PRL mask. A set of 180 evenly spaced virtual lines were cast radially outwards from the geometric centre of the lens. At each of the angular sampled positions, the distance to the nearest background pixel along each ray was recorded, and full-layer thickness was derived as twice this distance, scaled by the voxel size (1 µm/px). These values at each position were used to construct a 2D polar representation of PRL thickness across all 360 azimuthal slices. Maximum PRL thickness was calculated after removing the upper 1% of thickness values to reduce segmentation artefacts at layer boundaries.

To quantify spatial regionalisation of PRL thickness across developmental stages, mean maximum thickness was calculated within three anatomical zones: the HAZ (100-180°), the dorsal region (320-40°), and the nasal region (240-320°). The HAZ window was positioned over the temporo-ventral retina, consistent with the established localisation of the zebrafish high-acuity area (Yoshimatsu et al., 2020; Hernández-Bejarano et al., 2022). All three windows were set to an identical angular width of 80° so that the inter-regional thickness ratios were not biased by differences in the sampled area. Ratios of mean maximum thickness were calculated between the HAZ and dorsal, HAZ and nasal, and nasal and dorsal regions to assess the degree of regionalisation at each stage.

### Growth rate

Volumetric measurements for lens, PRL, and eye were performed on the same segmented radially resliced stacks, which were used for thickness measurement. For each slice, the volume of the tissue was calculated using a cylindrical Jacobian method. Each tissue pixel was treated as a small, curved wedge of a cylinder, and the volume contribution of each such pixel was calculated as dV=r·dx·dy·dθ. In this expression, r is the radial distance of each pixel from the axis of rotation (defined as the vertical axis passing through the geometric centre of the lens), dx and dy are the pixel dimensions (both equal to the isotropic voxel size of 1.0 µm), and dθ is the angular width of each slice (1°, expressed in radians as 2π/360). Summing these contributions across all tissue pixels in each slice gives the volume of that 1° wedge of tissue. The total volume of each structure (PRL, eye, or lens) was then obtained by summing wedge volumes across all 360 slices. For sector-level analysis, the same summation was restricted to the slices falling within each 10° angular window, yielding 36 sector volumes per structure per eye.

The SGR between consecutive developmental stages was calculated as SGR=(ln(V_2_)−ln(V_1_))/Δdays (unit: day^−1^), where V_1_ and V_2_ are the mean volumes at successive stages. SGR values were derived from the mean total volumes or sector volume of the structure (lens, PRL, and eye) per stage, and no per-fish SGR values were calculated owing to the cross-sectional design. The SGR is reported descriptively without statistical comparison between phases or sectors.

The axial length of the eye was calculated as the average distance between the anterior lens surface and the posterior pole of the eye, while the lens diameter was obtained as a mean of per-slice measures for an eye. Per-fish values were used to compute the ratio of lens diameter to axial length.

### Eye shape analysis

The eye radius for each radial slice was calculated as the maximum radial distance from the lens centre to the farthest eye boundary. These distances were used to generate a full circumferential radius profile per eye. Maximum diameter was calculated as the largest value obtained by summing the radii of two diametrically opposite slices (position *n* and 180+*n*).

To assess eye asymmetry at each developmental stage, radii at dorsal (0°), temporal (90°), ventral (180°) and nasal (270°) were extracted per eye. T/N, D/V, and DV/NT ratios were calculated per eye. T/N and D/V ratios were determined from the radii obtained at these cardinal points. The DV/NT ratio was defined as the DV diameter divided by the NT diameter, where each diameter was calculated as the sum of the two opposing cardinal radii. All three ratios were compared against 1.0 at each developmental stage to determine whether significant asymmetry was present (see ‘Statistical analysis’ section). All masks, model files and scripts used are publicly available on Zenodo (Syed et al., 2026).

### Optical coherence tomography (OCT)

OCT images were captured using the Leica Envisu system. A 12×8 mm scan window, comprising 600×80 (A×B) scans, was centred on the fovea for image acquisition. Informed consent was obtained from all involved participants. Ethical approval was received from the research ethics committee [references: (1) 20/EM/0040, (2) 10/H0406/74, (3) 12/EM/0261].

### Statistical analysis

All statistical analyses were performed in Python 3.12 (using SciPy 1.13.1 and NumPy 1.26.4). The unit of analysis was the individual fish, with per-fish values derived as the mean of left and right eyes. Where one eye was unavailable (as in one 5 dpf sample), the single available eye was used as the fish-level value.

Non-parametric tests were used throughout owing to small sample sizes (*N*=6-9 fish per stage). To assess whether measurements changed significantly across the five developmental stages, a Kruskal-Wallis test was applied as an omnibus test, followed by pairwise Mann-Whitney *U* tests between adjacent stage pairs. *P*-values from pairwise comparisons were corrected for multiple comparisons using the BH false discovery rate procedure. To assess whether ratio values differed significantly from a reference value of 1.0, one-sample Wilcoxon signed-rank tests were performed per stage. These ratio tests were not corrected for multiple comparisons, as each stage represents an independent biological question. All tests were two sided. Significance thresholds were set at *P<0.05, **P<0.01, and ***P<0.001.

## Supplementary Material



10.1242/biolopen.062760_sup1 Supplementary information
