# Supplementary material for: Development of the zebrafish foveal analogue: a quantitative atlas of high-acuity zone growth and retinal regionalisation
Source: Biol Open. 2026 Sep 1;15(9):bio062760. doi: 10.1242/bio.062760 (PMC13580590; doi:10.1242/bio.062760)
Supplement: Supplementary file 1 [file biolopen-15-062760-s1.pdf]

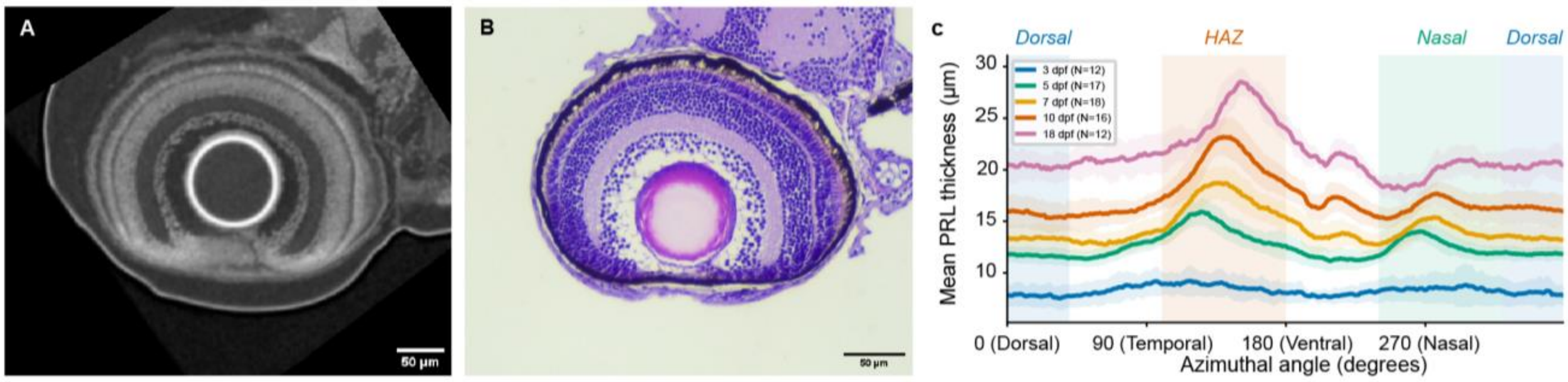

Fig. S1. Representative micro-CT and histological sections, and mean PRL thickness per stage. (A) Sagittal micro-CT section and (B) haematoxylin and eosin-stained paraffin section through 5 dpf wild-type eyes. The micro-CT volume was rotated to approximate the histological sectioning plane. Panels show different animals and are for qualitative representation only. Scale bars = 50 µm. **(C)** Azimuthal profile plot of mean PRL thickness across the eye at each developmental stage. Lines represent stage average; shaded bands indicate ± 1 SD. Vertical shaded regions denote the HAZ (orange; 100-180°), Nasal (green; 240-320°), and Dorsal (blue; 320-40°) reference zones. N indicates the number of eyes per stage. PRL: photoreceptor layer.

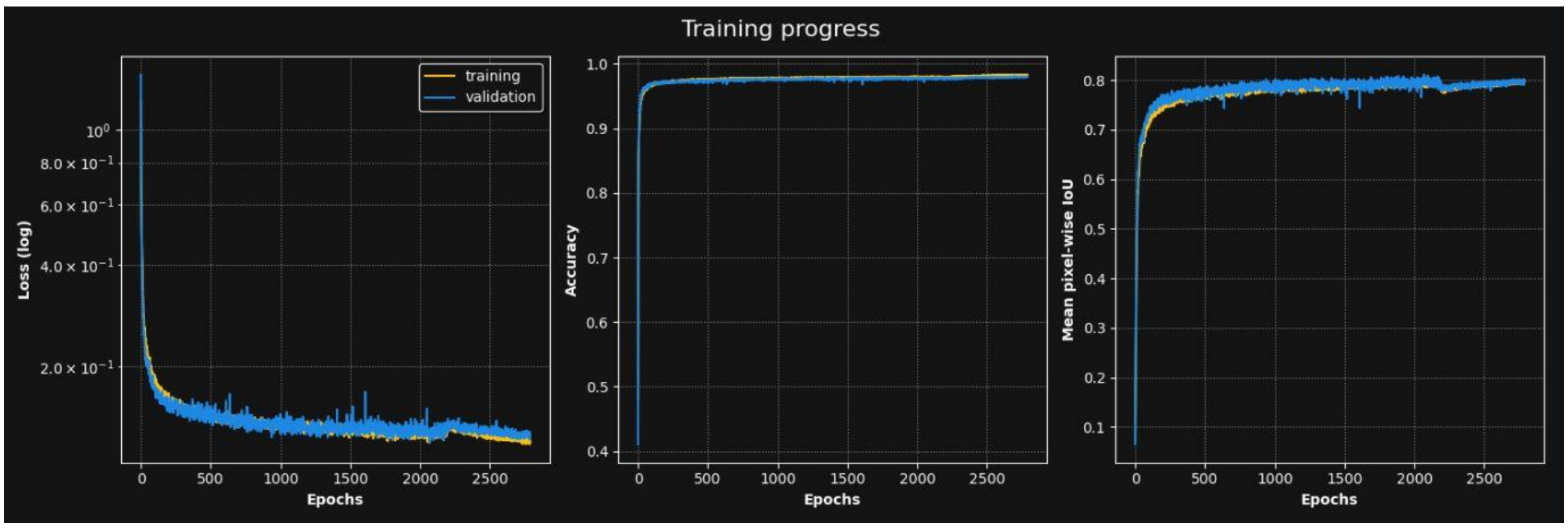

Fig. S2. Training performance of ARIVIS Cloud segmentation model used for PRL and lens segmentation. (A) Training and validation loss (log scale) across ~ 2,800 epochs. (B) Pixel-wise classification accuracy for training and validation sets for all the retinal layers and the lens. (C) Mean pixel-wise intersection over union (IoU). Traces are shown for the training (orange) and validation (blue) subsets. Training and validation traces remain closely overlapping throughout. Final validation pixel accuracy ~ 0.98 and mean pixel-wise IoU ~ 0.80. (PRL: photoreceptor layer)

Table S1. The composition of 77 manually annotated images used to train and validate the ARIVIS Cloud semantic segmentation model. Wild-type images span five developmental stages (3-18 dpf) and multiple eye quadrants. 46 additional 5 dpf images from morphologically distinct mutant larvae (from a separate study) were included to increase the robustness of model for a range of retinal morphology (dpf: days post fertilisation).

Developmental stage	Genotype / source	Annotated sections (n)
3 dpf	wild type	3
5 dpf	wild type	11
7 dpf	wild type	7
10 dpf	wild type	5
18 dpf	wild type	5
5 dpf	mutant (separate study)	46
**Total**	–	77

Table S2. Segmentation accuracy for the PRL and the lens on an independent held-out test set of 12 sections spanning all five developmental stages (3-18 dpf). These test images were excluded from both training and internal validation. Dice, IoU, precision and recall are computed from pooled pixel counts against manual ground-truth annotations, with background excluded. (PRL: photoreceptor layer)

Class	Dice	IoU	Precision	Recall
**PRL**	0.887	0.797	0.914	0.862
**Lens**	0.958	0.920	0.966	0.951

Table S3. Per-stage Dice and IoU for PRL and lens on the held-out test set, pooled by developmental stage. Segmentation accuracy for photoreceptor layer and lens by developmental stage.

Stage	PRL Dice	PRL IoU	Lens Dice	Lens IoU
**3 dpf**	0.883	0.791	0.958	0.919
**5 dpf**	0.896	0.811	0.954	0.912
**7 dpf**	0.920	0.853	0.976	0.953
**10 dpf**	0.870	0.769	0.979	0.958
**18 dpf**	0.882	0.789	0.947	0.899

Biology Open • Supplementary information

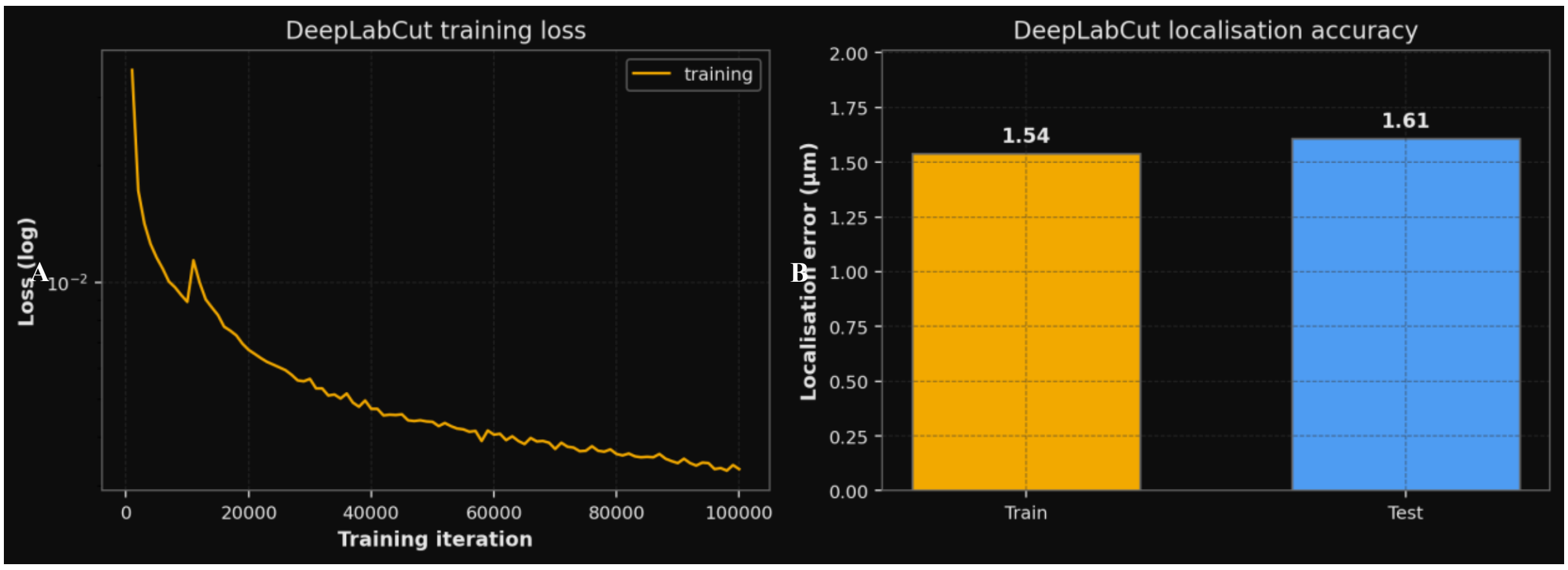

Fig. S3. DeepLabCut eye boundary model training and localisation accuracy. Training performance of the DeepLabCut model (12 peripheral keypoints) used to delineate the eye boundary. (A) training loss (log scale) over 100,000 iterations, converging to ~ 0.0033; the transient rise near 10,000 iterations coincides with the scheduled learning-rate increase (0.005 to 0.02). (B) localisation accuracy, quantified as the mean Euclidean distance between predicted and manually annotated keypoints on the training (95%) and held-out test (5%) frames, was 1.54 and 1.61 px respectively (1.54 and 1.61 µm at 1 µm/pixel). The model was trained on 200 manually labelled frames (40 per developmental stage) from 3, 5, 7, 10 and 18 dpf.

Table S4. Maximum photoreceptor layer (PRL) thickness across retinal quadrants across five zebrafish developmental stages.

Retinal quadrant		PRL thickness (µm)				
		3	5	7	10	18
Dor-Tem (0-90°)	Mean (95% CI)	10.1 (9.1-11.2)	14.9 (14.4-15.3)	16.4 (15.5-17.3)	18.9 (17.9-20.0)	25.1 (24.3-25.8)
	Max (95% CI)	11.7 (10.7-12.8)	16.7 (16.2-17.2)	18.1 (17.0-19.2)	20.5 (19.7-21.3)	27.3 (26.5-28.2)
Tem-Ven (90-180°)	Mean (95% CI)	11.1 (10.0-12.2)	19.0 (18.0-20.1)	23.4 (22.1-24.8)	27.9 (26.8-29.1)	34.1 (33.4-34.8)
	Max (95% CI)	12.3 (10.9-13.7)	25.6 (24.0-27.2)	29.9 (28.4-31.5)	37.1 (35.5-38.6)	45.7 (44.4-47.1)
Ven-Nas (180-270°)	Mean (95% CI)	10.5 (9.9-11.2)	15.3 (14.4-16.2)	18.9 (17.8-19.9)	22.7 (21.5-23.8)	27.8 (27.4-28.3)
	Max (95% CI)	12.0 (11.1-12.9)	18.2 (17.7-18.7)	22.8 (21.1-24.5)	27.6 (26.3-29.0)	34.4 (33.3-35.5)
Nas-Dor (270-360°)	Mean (95% CI)	10.6 (9.6-11.7)	15.6 (15.1-16.0)	17.2 (16.4-18.0)	19.8 (18.6-21.0)	25.2 (24.6-25.8)
	Max (95% CI)	12.0 (10.9-13.1)	18.0 (17.3-18.6)	19.9 (19.1-20.7)	22.1 (21.0-23.2)	28.1 (27.4-28.7)

Biology Open • Supplementary information

Maximum PRL thickness of each azimuthal slice was taken as the 99th percentile of its 180 radial measurements. Each per-fish value is the mean of paired left and right eyes. Each fish contributes either its average across the quadrant's 90 slices (Mean) or its single highest slice value (Max); both columns show the across-animal mean with 95% CI. Per-stage N: 3 dpf, n = 6 fish (12 eyes); 5 dpf, n = 9 fish (17 eyes); 7 dpf, n = 9 fish (18 eyes); 10 dpf, n = 8 fish (16 eyes); 18 dpf, n = 6 fish (12 eyes). Dor-Tem: Dorsal-Temporal; Tem-Ven: Temporal-Ventral; Ven-Nas: Ventral-Nasal; Nas-Dor: Nasal-Dorsal.

Table S5. Eye radius, whole-eye morphological dimensions, and lens mean radius across five zebrafish developmental stages**.**

**Retinal quadrant**		**Age (dpf)**				
		**3**	**5**	**7**	**10**	**18**
**Eye radius (µm)**						
**Dor-Tem (0-90°)**	Mean (95% CI)	122.1 (115.9-128.2)	133.4 (129.4-137.3)	148.0 (142.0-153.9)	154.1 (149.1-159.2)	187.7 (184.0-191.4)
	Max (95% CI)	128.9 (123.9-133.8)	140.8 (136.4-145.2)	155.1 (149.3-160.8)	162.5 (157.3-167.7)	195.6 (191.2-200.1)
**Tem-Ven (90-180°)**	Mean (95% CI)	126.5 (120.1-132.8)	138.1 (134.0-142.3)	155.0 (148.8-161.1)	168.9 (163.1-174.7)	214.6 (209.5-219.8)
	Max (95% CI)	132.3 (127.0-137.6)	150.9 (146.5-155.4)	164.4 (158.4-170.5)	180.0 (173.8-186.3)	228.9 (223.5-234.3)
**Ven-Nas (180-270°)**	Mean (95% CI)	131.5 (123.1-139.9)	132.0 (127.8-136.3)	143.5 (137.4-149.6)	151.2 (145.6-156.8)	187.9 (183.8-191.9)
	Max (95% CI)	140.6 (133.6-147.7)	150.1 (145.2-154.9)	157.9 (151.6-164.3)	160.0 (154.4-165.6)	203.1 (197.8-208.4)
**Nas-Dor (270-360°)**	Mean (95% CI)	129.3 (123.3-135.4)	140.5 (135.5-145.5)	154.5 (148.3-160.8)	157.1 (151.1-163.0)	194.6 (190.5-198.6)
	Max (95% CI)	140.6 (134.0-147.2)	150.6 (145.3-155.8)	163.0 (156.4-169.5)	163.9 (157.7-170.0)	200.3 (195.0-205.6)
**Whole-eye morphology (µm)**						
**Max diameter**	Mean (95% CI)	271.0 (259.5-282.4)	298.7 (289.0-308.3)	326.7 (313.3-340.1)	341.6 (329.4-353.8)	428.3 (418.5-438.1)
**Axial length**	Mean (95% CI)	157.3 (147.2-167.3)	168.7 (164.4-173.0)	194.2 (184.3-204.0)	202.4 (195.6-209.1)	257.3 (251.2-263.3)
**Lens mean radius (µm)**						
**Lens mean radius**	Mean (95% CI)	35.6 (31.6-39.6)	50.1 (48.0-52.2)	58.9 (57.4-60.5)	66.9 (64.3-69.5)	85.1 (82.9-87.2)

Biology Open • Supplementary information

Each per-fish value is the mean of paired left and right eyes. For the quadrant columns, each fish contributes either its average across the quadrant's 90 slices (Mean) or its single highest slice value (Max); both are reported as the across-animal mean with 95% CI. Maximum diameter (the largest diameter across an eye's meridians), axial length (distance from the anterior lens surface to the posterior pole) and lens mean radius (half the mean lens diameter) are whole-eye measurements, also reported as the across-animal mean with 95% CI. Per-stage N: 3 dpf, n = 6 fish (12 eyes); 5 dpf, n = 9 fish (17 eyes); 7 dpf, n = 9 fish (18 eyes); 10 dpf, n = 8 fish (16 eyes); 18 dpf, n = 6 fish (12 eyes). Dor-Tem: Dorsal-Temporal; Tem-Ven: Temporal-Ventral; Ven-Nas: Ventral-Nasal; Nas-Dor: Nasal-Dorsal.
