# Supplementary material for: Development of the zebrafish foveal analogue: a quantitative atlas of high-acuity zone growth and retinal regionalisation
Source: Biol Open. 2026 Sep 1;15(9):bio062760. doi: 10.1242/bio.062760 (PMC13580590; doi:10.1242/bio.062760)
Supplement: Supplementary file 2 [file biolopen-15-062760_review_history.pdf]

Development of the zebrafish foveal analogue: a quantitative atlas of high-acuity zone growth and retinal regionalisation

Hammad Syed, Manjiri Patil, William Jakins, Ha-Jun Yoon, Natalie Allcock, Gareth Douglas, William H.J. Norton and Mervyn Thomas

10.1242/bio.062760

Editor: Tristan Rodríguez

Review timeline
Original submission: 22 June 2026
Editorial decision: 1 July 2026
First revision received: 31 July 2026
Accepted: 3 August 2026

Original submission

First decision letter

MS ID#: bio.062760

MS Title: Development of the zebrafish foveal analogue: a quantitative atlas of high-acuity zone growth and retinal regionalisation

Authors: Hammad Syed, Manjiri Patil, William Jakins, Ha-Jun Yoon, Natalie Allcock, Gareth Douglas, William H.J. Norton and Mervyn Thomas

I have now reached a decision on the above manuscript.

The reviewer reports are shown at the bottom of this email.

As you will see, the reviewers raised a number of substantial criticisms that prevent me from accepting the paper at this stage.

They suggest, however, that a revised version might prove acceptable, if you can address their concerns. These primarily relate to providing further details on the segmentation method used and adjusting the claims of the manuscript so that they are more circumspect, and more accurately reflect the results that are presented in the manuscript. If you think that you can deal satisfactorily with the criticisms on revision, I would be pleased to see a revised manuscript.

At this stage, we also ask you to ensure your manuscript complies with our formatting guidelines. Provided you are able to fully address the referees' comments, we are positive about publication of your paper (we accept over 95% of revision submissions) and therefore hope you won't mind any extra work involved in reformatting your manuscript at this point.

Please upload both a 'clean' version of your Word file, along with a highlighted version clearly showing where you have made changes in the revised manuscript. Please avoid using 'Track changes' in Word files as these are lost in PDF conversion.

I should be grateful if you would also provide a point-by-point response detailing how you have dealt with the points raised by the reviewers in the 'Response to Reviewers' box. Please attend to

© 2026. Published by The Company of Biologists under the terms of the Creative Commons Attribution License (https://creativecommons.org/licenses/by/4.0/).

all of the reviewers' comments. If you do not agree with any of their criticisms or suggestions please explain clearly why this is so.

Reviewer 1

Comments for the author

The The authors use microCT data from fixed zebrafish samples to catalogue the development of the zebrafish analogue to the human fovea, the high acuity zone.

The study is interesting however does need some clarifications:

Experimental Quality
The authors use an automated segmentation tool; a comment on the segmentation error rate would be useful.
Do the authors have a measure of the shrinkage caused by fixation and processing? Were the authors able to compare thickness of, for example, the PRL, INL with live samples or from sections? I think for an atlas, understanding whether shrinkasge is isotrpoic or not would be necessary.

2. Reproducibility
Raw data has not been made available. Sharing the raw images and the segmentation is necessary. I think the statistical tests are fine, but I am not able to comment on them in too much depth. Human data (shown in Figure 4) needs an ethical statement and some methodological detail on its collection.

3. Completeness
if the shrinkage and segmentation checks are done, then this data is complete.

4. Scholarship.
- This is fine. Reference list needs some housekeeping!

Reviewer 2

Comments for the author

The manuscript by Syed and colleagues presents a contrast-enhanced micro-CT based quantitative atlas of zebrafish eye development, focusing on photoreceptor layer growth, lens and eye morphology and the emergence of a temporo-ventral high-acuity zone-like specialisation. This is a technically strong and valuable study that addresses a clear need for stage-resolved quantitative reference data in zebrafish retinal development. The imaging approach is appropriate, the sample size is reasonable for this type of 3D morphometric analysis and the main figures generally support the central morphological conclusions. The main issues with the current version of the manuscript concern data availability, segmentation validation and the need to keep mechanistic and translational claims aligned with the data presented.

Major points

- the manuscript is framed as a quantitative atlas and community resource but I could not identify clear deposition of the underlying data, segmentation masks, trained models, per fish measurements or analysis scripts. For an atlas paper, this is important. The authors should deposit the data underlying each figure, the analysis code used for PRL thickness, volume and growth-rate analyses and representative raw or processed micro-CT datasets. If the full raw imaging dataset is too large to deposit, the authors should at least provide complete per-fish measurements, processed masks and representative volumes.

- the segmentation pipeline is central to the study but needs stronger validation. The authors describe custom arivis-cloud and deeplabcut models followed by manual inspection but they do not report quantitative performance metrics. They should make clear how many images were used for training, validation and testing, whether test data were independent across stages and how segmentation performance was assessed.

© 2026. Published by The Company of Biologists under the terms of the Creative Commons Attribution License (https://creativecommons.org/licenses/by/4.0/).

-several mechanistic claims go beyond the presented data. The study convincingly shows regional PRL thickening and changes in eye, lens and PRL morphology but it does not directly measure photoreceptor number, subtype composition, cone packing, outer-segment length or cell recruitment. Therefore, claims that PRL thickening reflects photoreceptor packing, UV cone enrichment, segment elongation or recruitment should either be supported by additional validation or toned down. The data support progressive PRL thickening in the temporo-ventral retina but they do not establish the underlying cellular mechanism.

- the authors should be more precise in how they define and discuss the HAZ. Since the HAZ is usually defined by photoreceptor subtype distribution, ganglion cell density and functional specialisation, PRL thickness alone should be presented as a proxy. A more careful framing would be that the authors identify progressive PRL thickening in a temporo-ventral region corresponding to the expected HAZ.

-the "four growth phases" model is useful but it should be presented as a descriptive framework unless the authors add formal statistical testing of growth rate differences. Specific growth rates are calculated from stage mean volumes rather than per fish values so the SGR analysis does not support comparisons between phases or compartments in the same way as the per fish morphometric analyses.

- the human fovea comparison is interesting and figure 4 is useful as a conceptual visual comparison, especially because the legend states that the human and zebrafish stages are not temporally equivalent. However, the discussion should avoid implying that the same cellular mechanisms drive human foveal maturation and zebrafish HAZ development unless additional data are provided.

Minor points

-the authors should justify the anatomical boundaries used for HAZ, dorsal and nasal regions.

- the optic nerve head is aligned at approximately 220 degrees. The authors should report how much this position varied across samples and whether alignment variability could affect sector analyses, particularly the claim that the thickened region extends towards the optic nerve.

-The authors should clarify whether developmental stages included multiple clutches/breeding pairs and whether any clutch effects were considered.

Reviewer's Responses to Questions

Experimental quality
Are experiments performed using appropriate methods that will answer the question (or test the hypothesis or support the observations) posed by the authors? Is the right tool used for the job?
If 'No', please indicate reasons in Comments for Author box below.
Reviewer #1:

- No

Reviewer #2:

- Yes

Were the data analyzed using appropriate statistical tests?
If 'No', please indicate reasons in Comments for Author box below.
Reviewer #1:

- Yes

Reviewer #2:

- Yes

© 2026. Published by The Company of Biologists under the terms of the Creative Commons Attribution License (https://creativecommons.org/licenses/by/4.0/).

Reproducibility
Were experiments performed using adequate number of biological replicates?
If 'No', please indicate reasons in Comments for Author box below.
Reviewer #1:
- Yes

Reviewer #2:
- Yes

Is there sufficient raw data to assess the rigor of the analysis?
If 'No', please indicate reasons in Comments for Author box below.
Reviewer #1:
- No

Reviewer #2:
- No

Does the methods section provide sufficient detail to permit reproducibility?
If 'No', please indicate reasons in Comments for Author box below.
Reviewer #1:
- No

Reviewer #2:
- Yes

Completeness
Are the manuscript's conclusions supported by the data?
If 'No', please indicate reasons in Comments for Author box below.
Reviewer #1:
- Yes

Reviewer #2:
- No

Scholarship
Do the authors cite and discuss the merits of data that would argue for and against their conclusion?
If 'No', please indicate reasons in Comments for Author box below.
Reviewer #1:
- No

Reviewer #2:
- Yes

Does the manuscript title & abstract accurately reflect the contents of the manuscript, without hyperbole?
If 'No', please indicate reasons in Comments for Author box below.
Reviewer #1:
- Yes

Reviewer #2:
- Yes

© 2026. Published by The Company of Biologists under the terms of the Creative Commons Attribution License (https://creativecommons.org/licenses/by/4.0/).

First revision

Author response to reviewers' comments

Dear editors,

We are hereby submitting the revised manuscript "Development of the zebrafish foveal analogue: a quantitative atlas of high-acuity zone growth and retinal regionalisation" for consideration of publication in Biology Open.

We thank editor and the reviewers for their helpful feedback, and we have addressed their comments in our revised manuscript. The point-by-point response to the comments are added to this letter.

Point-by-point response

REVIEWER 1 COMMENTS

Sr. No.	Reviewer comments	Response	Location
1.	The authors use an automated segmentation tool; a comment on the segmentation error rate would be useful.	We thank the reviewer for raising this important point. Details about the segmentation error quantification can be found below:<br>Segmentation accuracy for photoreceptor layer (PRL) and lens was quantified on an independent held-out test set of 12 images spanning all five developmental stages, excluded from both training and internal validation. Against manual ground-truth annotations, the automated model achieved a Dice coefficient of 0.887 for the photoreceptor layer and 0.958 for the lens (Supplementary Table S2), corresponding to a low segmentation error for both. Accuracy was consistent across stages, with PRL Dice between 0.87 and 0.92 and lens Dice between 0.95 and 0.98 (Supplementary Table S3), indicating that no developmental stage was disproportionately affected. The whole-eye boundary DeepLabCut (DLC) model reached a mean localisation error of 1.54 px (training) and 1.61 px (held-out), i.e. 1.54–1.61 µm at 1 µm/pixel (Supplementary Figure S3). In addition, all masks used for	**Supplementary files:**<br><br>Supplementary Table S2 and S3.<br><br>**Methods:**<br><br>Line 432- 434<br>Line 446-447<br><br>**Data and Resource Availability:**<br><br>Line 537-546

© 2026. Published by The Company of Biologists under the terms of the Creative Commons Attribution License (https://creativecommons.org/licenses/by/4.0/).

		analysis were visually inspected in Dragonfly and manually corrected where necessary before measurement, so the residual error in the analysed masks is lower than the automated figures. These metrics have been added to the Supplementary Information and the held-out test images are deposited in Zenodo (DOI: 10.5281/zenodo.21415264). The method section also states the held-out images split now.	
2.	Do the authors have a measure of the shrinkage caused by fixation and processing? Were the authors able to compare thickness of, for example, the PRL, INL with live samples or from sections? I think for an atlas, understanding whether shrinkage is isotrpoic or not would be necessary.	We thank the reviewer for raising this point, which we agree is important to address explicitly for an atlas dataset.<br><br>A direct, per-layer measurement of processing-related shrinkage is not obtainable within our pipeline. Micro-CT imaging and histological sectioning are mutually exclusive terminal procedures: once an animal has been fixed, contrast-stained, dehydrated, and resin-embedded for micro-CT, it cannot subsequently be paraffin-sectioned, and a sectioned specimen cannot be imaged volumetrically. A matched within-animal comparison of PRL or INL thickness across the two modalities is therefore not achievable, and we cannot provide a quantitative shrinkage factor or to determine whether any dimensional change is isotropic.<br><br>We did carry out an exploratory comparison of lens diameter between micro-CT and histological paraffin sections from a small separate cohort of animals at 3, 5, and 7 dpf (n = 4 fish per stage for histology). Differences between the two methods were modest and showed no consistent direction across	**Supplementary files:**<br><br>Supplementary Figure 1A and 1B.<br><br>**Discussion:**<br><br>Line 351-361

© 2026. Published by The Company of Biologists under the terms of the Creative Commons Attribution License (https://creativecommons.org/licenses/by/4.0/).

		stages. However, we did not consider this comparison sufficiently robust to present as a formal validation: the two cohorts comprise different individuals, paraffin processing introduces its own shrinkage and therefore does not provide an unshrunken reference, and 5 µm sectioning may not capture the lens equator, systematically biasing the histological maximum. We have therefore not included these measurements in the manuscript but are happy to provide them to the reviewer if that would be helpful.<br><br>While we cannot quantify shrinkage in our own specimens, published work using a comparable pipeline provides some reassurance regarding dimensional fidelity. Ding et al. (2019) used a broadly similar contrast-enhanced micro-CT preparation in larval zebrafish and validated it against an independent standard, recovering sarcomere spacings that matched published values and cross-sections that closely resembled conventional histology. This suggests that preparations of this type preserve cellular-scale dimensions well, though it is not a direct measure of shrinkage or isotropy in our own material. We have also included a comparative figure for micro-CT and histology sagittal sections to further highlight the similarity and differences of the two approaches.<br><br>Ultimately, absolute dimensions from any fixed-tissue modality are method-dependent, which is why we have built the atlas around relative rather than absolute measures. Every specimen in this study underwent identical processing, so whatever dimensional change occurs	

© 2026. Published by The Company of Biologists under the terms of the Creative Commons Attribution License (https://creativecommons.org/licenses/by/4.0/).

		applies uniformly across stages and animals.<br><br>We have revised the limitations section of the Discussion, accordingly, stating that per-layer shrinkage was not measured and isotropy was not assessed, that absolute values should be read as specific to this preparation, and that the ratio- and trajectory-based conclusions are robust to uniform scaling.	
3.	Raw data has not been made available. Sharing the raw images and the segmentation is necessary.	Same as Reviewer 2, comment 1.	
4.	Human data (shown in Figure 4) needs an ethical statement and some methodological detail on its collection.	We thank the reviewer for pointing this out. We have added an ethical statement and methodological details in the Methods section now.<br><br>*"Optical Coherence Tomography (OCT) images were captured using the Leica Envisu system. A 12 x 8 mm scan window, comprising 600 X 80 (A X B) scans, was centered on the fovea for image acquisition. Ethical approval was received from the research ethics committee (REC references: (1) 20/EM/0040, (2) 10/H0406/74, (3) 12/EM/0261)."*	**Methods:**<br><br>Line 510-514
5.	Reference list needs some housekeeping!	We thank the reviewer for pointing this out. We have reviewed our Reference list and have made the necessary Changes.	

REVIEWER 2 COMMENTS

Sr. No.	**Reviewer comments**	Response	Location
1.	the manuscript is framed as a quantitative atlas and community resource but I could not identify clear deposition of the underlying data, segmentation masks, trained models, per fish measurements or analysis scripts. For an atlas paper, this is important. The authors should deposit the data	We thank both the reviewers and have now deposited a comprehensive data and code record in Zenodo (DOI: 10.5281/zenodo.21415264), comprising: the segmentation masks for the photoreceptor layer, lens and whole eye; the complete per-fish measurements underlying every	**Data and Resource Availability:**<br><br>Line 537-546<br><br>**Methods:**<br><br>Line 507

© 2026. Published by The Company of Biologists under the terms of the Creative Commons Attribution License (https://creativecommons.org/licenses/by/4.0/).

	underlying each figure, the analysis code used for PRL thickness, volume and growth-rate analyses and representative raw or processed micro-CT datasets. If the full raw imaging dataset is too large to deposit, the authors should at least provide complete per-fish measurements, processed masks and representative volumes.	figure; the full analysis code for PRL thickness, volume and growth-rate analyses; both trained segmentation models (arivis Cloud and DeepLabCut) with configuration files; and the held-out segmentation validation set and DeepLabCut labelled training frames, each with accompanying documentation, and representative volume videos. The raw micro-CT image volumes are not included due to their large size, and the deposited masks, per-fish measurements, models and code together reproduce every quantitative result in the manuscript. We have referenced this data in the Data and Resource Availability section and added a line in the Method section.	
2.	the segmentation pipeline is central to the study but needs stronger validation. The authors describe custom arivis-cloud and deeplabcut models followed by manual inspection but they do not report quantitative performance metrics. They should make clear how many images were used for training, validation and testing, whether test data were independent across stages and how segmentation performance was assessed.	We thank the reviewer and have added a full quantitative validation. (We have addressed it in comment 1 for Reviewer 1).<br><br>The ARIVIS Cloud model was trained on 77 manually annotated images spanning all five developmental stages and multiple retinal quadrants, of which 46 were from morphologically distinct mutant larvae included to increase robustness. Arivis Cloud partitioned these into internal training and validation subsets, with training/validation loss, pixel accuracy and mean IoU for all retinal layers (photoreceptor layer, lens and other retinal layers) monitored to convergence (~2,800 epochs; final validation pixel accuracy ~0.98, mean IoU ~0.80; Supplementary Figure S2). Performance was then assessed on a separate held-out test set of 12 wild-type images spanning all five stages (3-18 dpf), excluded from both training and internal validation. Per-class accuracy on this independent set is reported as Dice, IoU,	**Supplementary files:**<br><br>Supplementary Table S2 and S3.<br><br>**Methods:**<br><br>Line 425- 434<br>Line 440-447<br><br>**Data and Resource Availability:**<br><br>Line 537-546

© 2026. Published by The Company of Biologists under the terms of the Creative Commons Attribution License (https://creativecommons.org/licenses/by/4.0/).

		precision and recall (Supplementary Table S2), with per-stage breakdown confirming consistency across development (Supplementary Table S3). The whole-eye boundary (DeepLabCut) model was trained on 200 labelled frames (40 per stage) with a 95%/5% train/test split over 100,000 iterations, reaching 1.54/1.61 px localisation error (Supplementary Figure S3). The test set is thus independent of training data and spans every developmental stage.	
3.	several mechanistic claims go beyond the presented data. The study convincingly shows regional PRL thickening and changes in eye, lens and PRL morphology but it does not directly measure photoreceptor number, subtype composition, cone packing, outer-segment length or cell recruitment. Therefore, claims that PRL thickening reflects photoreceptor packing, UV cone enrichment, segment elongation or recruitment should either be supported by additional validation or toned down. The data support progressive PRL thickening in the temporo-ventral retina but they do not establish the underlying cellular mechanism.	We thank the reviewer for this valuable point and agree that our data establish progressive regional PRL thickening but do not directly resolve the underlying cellular mechanisms. We have revised the Discussion throughout to ensure that all cellular interpretations are clearly attributed to the existing literature and framed as consistent with, rather than demonstrated by, our measurements.	**Discussion:**<br><br>Line 250-252<br>Line 259-262<br>Line 284-285<br>Line 287-289<br>Line 326-330
4.	the authors should be more precise in how they define and discuss the HAZ. Since the HAZ is usually defined by photoreceptor subtype distribution, ganglion cell density and functional specialisation, PRL thickness alone should be presented as a proxy. A more careful framing would be that the authors identify progressive PRL thickening in a temporo-ventral region corresponding to the expected HAZ.	We thank the reviewer for this important point and agree that PRL thickness alone does not fully define the HAZ. We have revised the manuscript to mention PRL thickness explicitly as a structural proxy for HAZ specialisation and to frame our findings as progressive PRL thickening in the temporoventral region corresponding to the HAZ. We have made changes in the introduction, discussion and results section to indicate that the PRL thickness is a structural proxy of HAZ.<br>We believe this framing more accurately reflects what our measurements establish while	**Summary:**<br><br>Line 37<br><br>**Introduction:**<br><br>Line 106<br>Line 128<br>Line 132-133<br><br>**Result:**<br><br>Line 139<br>Line 207<br><br>**Discussion:**<br><br>Line 241-242<br>Line 246

© 2026. Published by The Company of Biologists under the terms of the Creative Commons Attribution License (https://creativecommons.org/licenses/by/4.0/).

		retaining the biological relevance of the temporo-ventral thickening we observe.	
5.	the "four growth phases" model is useful but it should be presented as a descriptive framework unless the authors add formal statistical testing of growth rate differences. Specific growth rates are calculated from stage mean volumes rather than per fish values so the SGR analysis does not support comparisons between phases or compartments in the same way as the per fish morphometric analyses.	We thank the reviewer for this important clarification and fully agree. We have revised the manuscript to present the specific growth rate as descriptive support to the per-fish volumetric measurements which is the basis of the four-phase model.<br><br>We would like to clarify the statistical basis of the phases, which we have now made explicit in the text. The phase boundaries are not defined by SGR but by statistically supported transitions in the per-fish PRL/eye volume fraction and the per-fish lens diameter to axial length ratio. Both of these were analysed using Kruskal-Wallis followed by pairwise Mann-Whitney U tests with Benjamini-Hochberg correction on per-fish values. Phases I and III correspond to significant increases in these per-fish ratios, while Phases II and IV correspond to non-significant transitions. The SGR values, as the reviewer correctly notes, are calculated from stage-mean volumes and are therefore now presented solely as a descriptive illustration of the underlying volumetric changes.<br><br>We would also note that per-fish SGR could not be computed in this study because different individual animals were imaged at each developmental stage. Longitudinal per-fish growth rates are therefore not obtainable from this dataset, and we have made this explicit in the Methods.<br><br>We believe these changes address the reviewer's concern while preserving the four-phase model as a useful descriptive framework grounded in our per-fish morphometric analyses.	**Summary:**<br><br>Line 33<br><br>**Results:**<br><br>Line 168<br>Line 176<br>Line 200-202<br><br>**Discussion:**<br><br>Line 264-266<br>Line 274-282<br><br>**Methods:**<br><br>Line 487-488<br><br>**Figure Legends:**<br><br>Line 586

© 2026. Published by The Company of Biologists under the terms of the Creative Commons Attribution License (https://creativecommons.org/licenses/by/4.0/).

6.	the human fovea comparison is interesting and figure 4 is useful as a conceptual visual comparison, especially because the legend states that the human and zebrafish stages are not temporally equivalent. However, the discussion should avoid implying that the same cellular mechanisms drive human foveal maturation and zebrafish HAZ development unless additional data are provided.	We thank the reviewer for this helpful comment and for noting the Figure 4 as a conceptual comparison. We agree that our data do not establish a shared cellular mechanism between human foveal maturation and zebrafish HAZ development, and we have revised the Discussion so that the comparison is framed at the level of the developmental pattern rather than the underlying cellular processes.<br><br>Where cellular mechanisms are mentioned, cone outer-segment elongation and packing in the zebrafish HAZ are now attributed to the studies that directly measured them, and we state explicitly that whether these processes continue to drive the later thickening we observe remains to be determined.	**Summary:**<br><br>Line 36-37<br><br>**Discussion:**<br><br>Line 317<br>Line 326-327<br>Line 329-330
	**Minor points**		
7.	the authors should justify the anatomical boundaries used for HAZ, dorsal and nasal regions.	We thank the reviewer for raising this. We have added a justification to the Methods.<br><br>*"The HAZ window was positioned over the temporo-ventral retina, consistent with the established localisation of the zebrafish high-acuity area (Yoshimatsu et al., 2020; Hernández-Bejarano et al., 2022). All three windows were set to an identical angular width of 80°, so that the inter-regional thickness ratios were not biased by differences in sampled area."*	**Methods:**<br><br>Line 464-469
8.	the optic nerve head is aligned at approximately 220 degrees. The authors should report how much this position varied across samples and whether alignment variability could affect sector analyses, particularly the claim that the thickened region extends towards the optic nerve.	We thank the reviewer for this comment. In addressing it we identified a transcription error: the optic nerve head reference position was incorrectly stated as ~220°. The correct value is ~200°, and this has been corrected in both the Methods and Results.<br><br>The optic nerve head was actively standardised using a	**Method**s:<br><br>Line 409-411<br>Line 416-420<br><br>**Results:**<br><br>Line 154

© 2026. Published by The Company of Biologists under the terms of the Creative Commons Attribution License (https://creativecommons.org/licenses/by/4.0/).

		two-stage alignment. An approximate angular alignment was achieved during the first orientation in Fiji, after which the raw radially resliced images and their corresponding masks were registered so that the optic nerve head was positioned at ~200° in every eye, to within the 1° reslicing resolution.<br><br>Because all analysis was done on the registered masks, alignment variability cannot bias the sector ratios or the observed ventral extension of thickening towards the optic nerve. We have revised the Methods to describe this two-stage alignment explicitly.	
9.	The authors should clarify whether developmental stages included multiple clutches/breeding pairs and whether any clutch effects were considered.	We thank the reviewer for this point. Embryos were obtained from group crosses (two males and two females per tank). Fish at each developmental stage were sampled from multiple clutches (two to four per stage), with no single clutch contributing more than half of the individuals at any stage. Clutch effects were therefore considered at the sampling-design stage and mitigated in this way. We have added this information to the Methods.	**Methods:**<br><br>Line 71-81

Second decision letter

MS ID#: bio.062760R1

MS Title: Development of the zebrafish foveal analogue: a quantitative atlas of high-acuity zone growth and retinal regionalisation

Authors: Hammad Syed, Manjiri Patil, William Jakins, Ha-Jun Yoon, Natalie Allcock, Gareth Douglas, William H.J. Norton and Mervyn Thomas

I am happy to tell you that your manuscript has been accepted for publication in Biology Open, pending our standard publication integrity checks. It was accepted on 3rd August 2026.

© 2026. Published by The Company of Biologists under the terms of the Creative Commons Attribution License (https://creativecommons.org/licenses/by/4.0/).
